# Comparison between standard hematological parameters and blood doping biomarkers in dried blood spots within the athlete population of Swiss Sport Integrity

**DOI:** 10.3389/fspor.2024.1452079

**Published:** 2024-09-19

**Authors:** Jessica Almeida Oliveira, Francesco Loria, Céline Schobinger, Tiia Kuuranne, Claudia Mumenthaler, Nicolas Leuenberger

**Affiliations:** ^1^ Swiss Laboratory for Doping Analyses, University Center of Legal Medicine, Lausanne, Switzerland; ^2^ Lausanne University Hospital & University of Lausanne, Lausanne, Switzerland; ^3^ Swiss Sport Integrity, Bern, Switzerland

**Keywords:** RNA biomarkers, dried blood spots (DBS), hematological module, Athlete Biological Passport (ABP), blood doping

## Abstract

**Introduction:**

The study demonstrated the feasibility of incorporating RNA biomarkers, specifically 5-aminolevulinic acid synthase (ALAS2) and carbonic anhydrase 1 (CA1), to improve the hematological module of the Athlete Biological Passport (ABP) in routine antidoping context.

**Objective:**

The aim was to investigate the implementation of reticulocyte (RET) related biomarkers, specifically ALAS2 and CA1, using quantitative reverse transcription polymerase chain reaction (RT-qPCR) on dried blood spots (DBS) from elite athletes. Hemoglobin changes over time in DBS samples was measured as well. Combining hemoglobin and messenger RNA (mRNA) analyses allowed to monitor alterations of the established marker, “DBS OFF-score”.

**Methodology:**

Ten athletes were selected for sampling by the Swiss national antidoping organization, Swiss Sports Integrity (SSI). Samples were collected, transported and analyzed for ABP following the World Anti-Doping Agency (WADA) procedures and spotted onto Protein Saver DBS cards.

**Results:**

Most athletes exhibited stable biomarker levels, except for one individual involved in ski mountaineering, who demonstrated a sustained increase in ALAS2 compared to the individual baseline. This elevation could be due to blood withdrawal or other factors, such as doping with substances outside the targeted test menu.

**Conclusion:**

In this study, RNA-biomarkers were successfully analyzed in routine blood samples, and the project demonstrated promising results for the implementation of ALAS2 and CA1 in routine analysis to complement the ABP.

## Introduction

In endurance sports, blood doping is one of the most used doping strategies to improve physical performance. It involves manipulating the erythrocyte level, to increase hemoglobin (HGB) mass in the body, through substances/methods banned by the World Anti-Doping Agency (WADA) such as the administration of recombinant human erythropoietin (rhEPO) or blood transfusions (1).

Actually, blood doping can be detected directly and indirectly via the Athlete Biological Passport (ABP) (2). The hematological module of the ABP longitudinally monitors various biomarkers such as reticulocytes percentages (%RET), hemoglobin concentration (HGB) and OFF-score $\left(\mathrm{OFF}-\mathrm{score}=(\mathrm{HGB}\;[g/L])-\left(60\times \sqrt{\text{\%}\mathrm{RET}}\right)\;\right)$ of an athlete, following the requirements established by WADA (3, 4). This approach allows for observing atypical blood results by analyzing intraindividual data over time. The individual tolerance ranges (upper and lower) are constructed by a Bayesian algorithm based on measured values in athlete's samples as well as population data, gender and age. The specificity of the method is set to 99% to identify atypical values (3, 5, 6). Samples are collected, transported, and analyzed before being assessed by the Athlete Passport Management Units (APMU) and evaluated by experts. APMUs are integrated within WADA accredited laboratories and serve as the liaison between the external experts and antidoping organizations (passport custodians) to manage the ABP (7–9). While the hematological ABP currently includes numerous blood parameters and additional information such as the age, gender and exposure to high altitudes to help in passport evaluation; some blood doping practices, such as micro-dosing with rhEPO, can be challenging to detect (1, 4, 10). Moreover, confounding factors such as hypoxic training, altitude residence, or excessive training complicate passport interpretations by introducing important variations between and within athletes (11).

The matrix used in ABP analysis is ethylenediaminetetraacetic acid (EDTA) blood tubes. Blood samples collected in EDTA tubes are analyzed with Sysmex XN instrument, which provides harmonized results of selected biomarkers between WADA accredited and approved laboratories. However, the main limitations associated with this matrix are related to transport and storage (12). In comparison with other antidoping samples, management of EDTA tubes presents challenges as prompt sample delivery, refrigeration, and temperature monitoring is required. The validity of a blood sample can be compromised by long delivery times and unacceptable temperatures, and a so-called blood stability score (BSS) has been developed to record this (13). Moreover, a fast cooling of the blood appears to negatively impact the cells and consequently affect ABP results analysis (12).

With the advancement in research techniques, transcriptomics has been explored for various purposes (14–16). Therefore, studies focusing on mRNA biomarkers specific to erythropoiesis have been conducted, revealing that some RET-related mRNA could serve as biomarkers for blood doping due to changes in their expression level (17–20). It has been demonstrated that mRNA extraction from dried blood spots (DBS) is efficient and biomarkers such as mRNA are even more preserved when the blood is completely dried (19). Regarding ABP parameters, HGB and OFF score could be measured on DBS as well (21). Moreover, DBS require lower blood levels, less storage space and appear to be less sensitive to storage conditions compared to EDTA blood tubes (18, 22). Indeed, the potential of the DBS sample to improve the reliability of the hematological ABP-module is largely related to the improved stability of the blood sample.

Two erythropoiesis-related mRNA biomarkers, *5-aminolevulinic acid synthase (ALAS2)* and *carbonic anhydrase 1 (CA1)*, have been identified as being sensitive to changes associated with blood doping. It appears that *ALAS2* and *CA1* are downregulated after a transfusion (20). Both targeted genes were also demonstrated to be downregulated during an ABT of approximately 280 ml of RBC, showing the highest decrease after 9 days (20). In addition, both biomarkers have been found sensitive to detect doping with EPO micro-doses, leading to an increase of more than 2.5× for *ALAS2* and nearly 2× for *CA1* (17, 19, 23). Furthermore, they seem to be less influenced by altitude compared to current biomarkers (17).

In this study, the practical application of RNA-biomarkers in DBS samples within an authentic context of routine samples was tested. Specifically, we evaluated samples of Swiss athletes, collected in whole blood EDTA tubes for the purpose of hematological module of the ABP.

## Material and methods

### Routine samples

**Figure 1:**
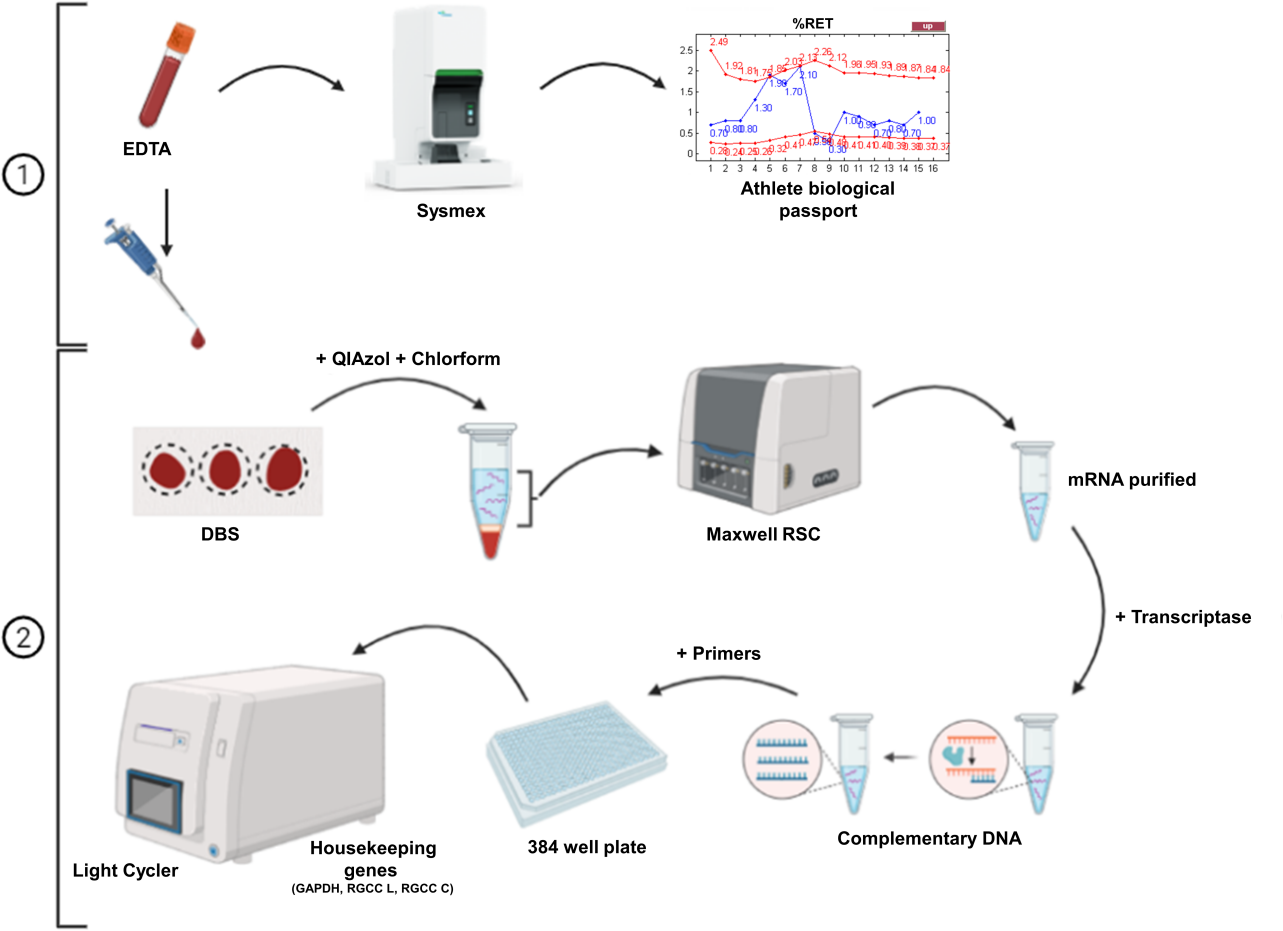
Scheme of routine experiment processing analysis of EDTA blood tubes. **(1)** Blood samples are analyzed using a Sysmex XN instrument, and the data is utilized for the Athlete Biological Passport (ABP). Blood from EDTA tubes is then spotted onto dried blood spot (DBS) cards. **(2)** Analysis of DBS: The DBS samples undergo RNA purification, followed by cDNA preparation and analysis using quantitative reverse transcription polymerase chain reaction (RT-qPCR).

Swiss Sport Integrity (SSI) team selected athletes to participate in this study. A pool of 10 high-level athletes (both male and female) practicing various endurance sport, where erythropoiesis enhancement could be used, was constructed by SSI and 3–5 out-of-competition EDTA samples were collected in monthly intervals for each athlete (Table 1). Written informed consent for participation was not required from the participants or the participants’ legal guardians/next of kin in accordance with the national legislation and institutional requirements because Athletes agreed for research consent on doping control form. EDTA samples were collected, transported and analyzed following routine WADA procedure (24). After analysis in the Sysmex XN, tubes were homogenized for 15 min and 20 µl of blood from EDTA samples were spotted in 903™ Protein Saver DBS Cards (Cytiva, USA) and stored at 4°C (Figure 1). DBS samples were used for RNA and hemoglobin analyses as described below.

**Table 1:** Athletes, gender/sport disciplines investigated in the study.

Athletes	Gender	Sport
3	Male	Skiing/Cross-Country
2	Female	Athletics/Long Distance (3,000 m or greater)
1	Male	Ski-Mountaineering
1	Female	Athletics/Middle Distance (800–1,500 m)
1	Male	Cycling road
1	Female	Cycling road
1	Male	Cycling/Track Endurance

### RET-related mRNA extraction and analysis in DBS

The process of the experiment is the same as described for previous studies (17, 25).

Each DBS was cut and put in into a 2 ml conical polypropylene microcentrifuge tube (Eppendorf, Switzerland). Lysin reagent phenol/guanidine (1 ml, Qiagen, Germany) was added to each tube and incubated shaking (15 min, 450 rpm at 37°C). Short centrifugation (8 s) was performed before sonication (15 min, Sonicator S30®, Elmasonic®, Germany). An addition of 250 μl of Chloroform was followed by another shaking incubation (15 min, 450 rpm at 37°C). Each tube was vortexed twice to then be incubated 5 min at room temperature and centrifuged 15 min at 10,000 rcf. The supernatant (525 μl) was transferred into Maxwell Cartridges followed by the manufacture instructions, to do an automatically RNA purification with a Maxwell® RSC Instrument (Promega, USA) with a Maxwell® RSC miRNA Plasma and Serum Kit. Elution tubes were recovered and stored at −80°C unless the complementary DNA (cDNA) preparation has been done the same day.

In 0.5 ml tubes (Eppendorf, Switzerland), 11 µl (50–100 ng) of purified mRNA was added to 9 µl of the mix composed of 4 µl of buffer, 2 µl of deoxynucleotide, 2 µl of hexamer, 0.5 µl RNase inhibitors and 0.5 µl of transcriptase prepared using Transcriptor First Stand cDNA Synthesis Kit (Roche, Switzerland). Non-Reverse transcription (NRT) control was added. Tubes were then incubated 5 min at 25°C, 10 min at 55°C and finally 5 min at 85°C. A short centrifugation of 8 s was performed to remove the drop on the lid and the cDNA containing tube was then placed at 4°C.

After the extraction and the cDNA preparation, RT-qPCR analysis was performed. Mean of three (3) housekeeping genes (*GAPDH*, *RGCC L*, *RGCC C*) were selected to normalize the results of the three (3) targeted mRNA (*ALAS2 L, ALAS2 LC, CA1)*. All samples were analyzed in triplicates for each gene. An aliquot of cDNA sample (4 µl) was loaded to a 384 well plate (Roche Life Science, City, Country), with primer mix (6 µl) that was composed of SYBR Green Master Mix (24 µl, Qiagen, Germany) and primers (MicroSynth, balgach, Switzerland). The plate was centrifugated at 2,000 rpm during 2 min before being analyzed by the Light Cycler 480 System (Roche Life Science, City, Country). Non-template control (NTC) was added. The programmed cycles of the Light Cycler were as follows: one cycle of denaturation of 10 min followed by 45 cycles of amplification 10 s all at 95°C and 1 min at 60°C; one cycle of melting curve of 1 min at 55°C and 5 s at 95°C and finally cooled down to 40°C during 30 s.

Relative quantification of *ALAS2* and *CA1* results were analyzed by the Light Cycler software (version 1.5.0.39), %RET results were analyzed with Sysmex XN instrument following analytical requirement for hematological module of the ABP (TD2021BAR) (26). To compare%RET and erythropoiesis related mRNA biomarkers, all data were compared to the baseline as percentage.

### HGB absorbance in DBS

The process of the experiment is the same as described for a previous study (21).

Each DBS was cut and put in into a 2 ml conical polypropylene microcentrifuge tube (Eppendorf, Switzerland). A volume of 1 ml of SDS 0.06% in Mili Q water was added to each tube and incubated shaking (15 min, 450 rpm at 37°C). Short centrifugation (8 s) was performed before sonication (15 min) (Sonicator S30®, Elmasonic®, Germany). Another shaking incubation (15 min, 450 rpm at 37°C) was performed and 200 μl of the content of the tube was added into ELISA plate well. Each sample was prepared in triplicates and the absorbance was calculated at 540 nm.

To be compared with the EDTA blood profiling data, quality control samples used for the Sysmex were processed and analyzed as well.

### OFF-score calculations

The OFF-score calculation for ALAS2 and CA1 biomarkers was adapted from the prevailing calculation of OFF-score, based on HGB DBS data and the%RET biomarker the HGB Sysmex data, replacing the%RET by RNA biomarker as described in (21).

$$\mathrm{OFF}\;\mathrm{SCORE}=(\mathrm{HGB}\;[g/L])-\left(60\times \sqrt{\mathrm{Biomarker}}\right)$$

## Results and discussion

The routine valid (BSS below 85 and no negative temperature) samples were received and analyzed before being spotted onto DBS cards (Figure 1). This method does not require additional sampling from the athlete and only necessitates a simple and quick additional preparatory stage upon sample analysis. Furthermore, due to the superior long-term stability of DBS compared to EDTA blood samples, an athlete's samples can be batched and analyzed together, thereby reducing variation between different analyses.

**Figure 2:**
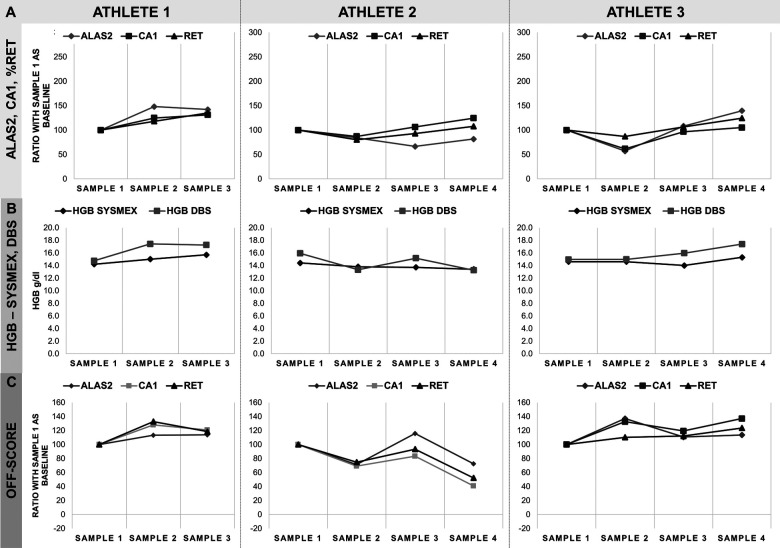
Monitoring of three athletes from cycling. **(A)** ALAS2, CA1 and%RET data as a percentage of the first sample, considered as baseline value, and corresponding 100% on the *y*-axis. **(B)** Comparison of HGB-values obtained in Sysmex-analysis and as calculated on the DBS. HGB data are represented in *y*-axis as g/dl. **(C)** Calculated OFF-score for all markers based on HGB,%RET, ALAS2 and CA1 data. ALAS2 is represented in dark grey diamonds (♦), CA1 in light grey squares (▪), %RET in black triangles (▴), HGB SYSMEX in black diamonds (♦) and HGB DBS in light grey squares (▪). Athlete 1: Cycling/Track Endurance (male); Athlete 2: Cycling/Road (female); Athlete 3: Cycling/Road (male). Samples were collected in monthly intervals.

**Figure 3:**
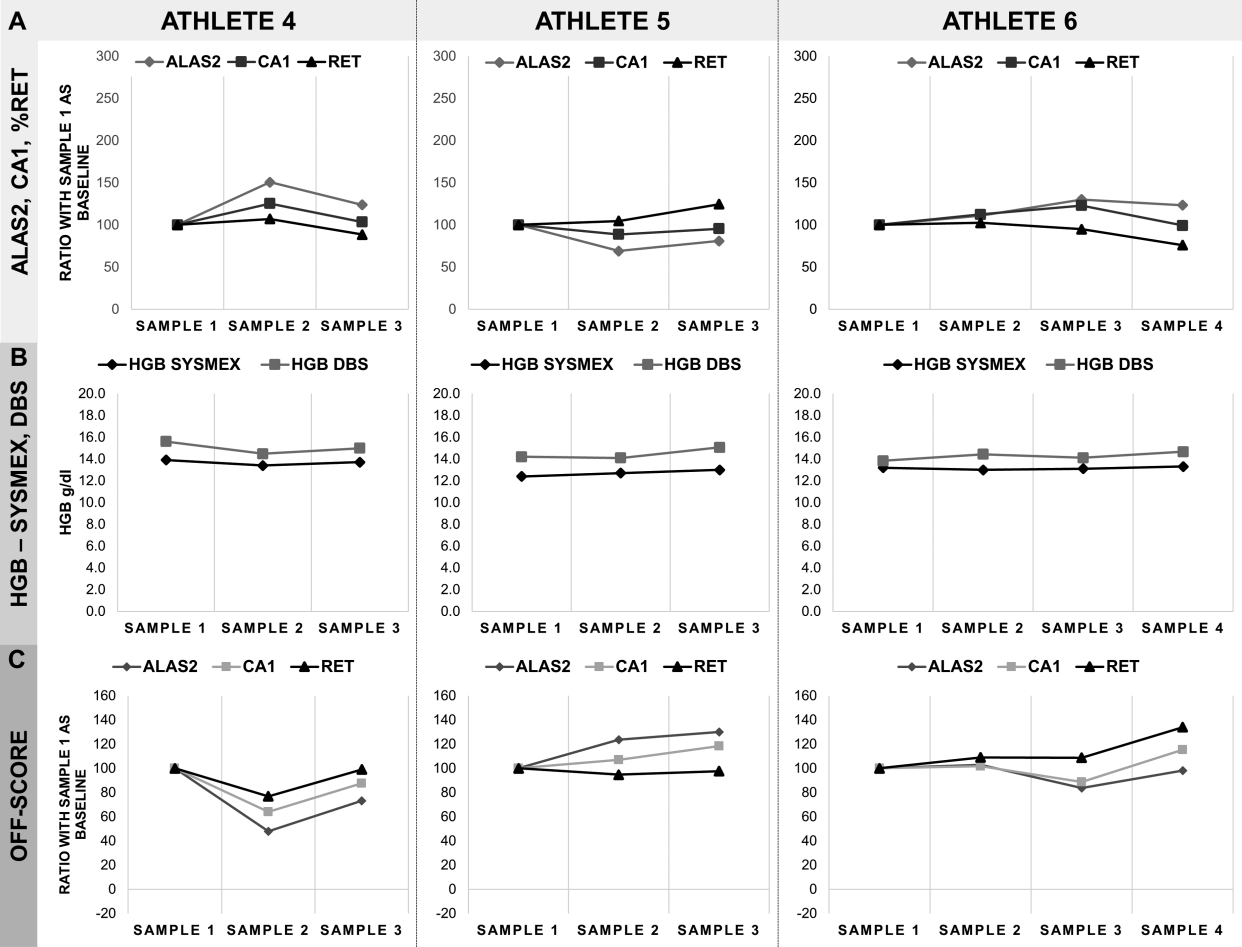
Monitoring of three athletes from athletics. **(A)** ALAS2, CA1 and%RET data as a percentage of the first sample, considered as baseline value, and corresponding 100% on the *y*-axis. **(B)** Comparison of HGB-values obtained in Sysmex-analysis and as calculated on the DBS. HGB data are represented in *y*-axis as g/dl. **(C)** Calculated OFF-score for all markers based on HGB,%RET, ALAS2 and CA1 data. ALAS2 is represented in dark grey diamonds (♦), CA1 in light grey squares (▪), %RET in black triangles (▴), HGB SYSMEX in black diamonds (♦) and HGB DBS in light grey squares (▪). Athlete 4: Athletics/Long distance (female); Athlete 5: Athletics/Middle distance (female); Athlete 6: Athletics/Long distance (female). Samples were collected in monthly intervals.

**Figure 4:**
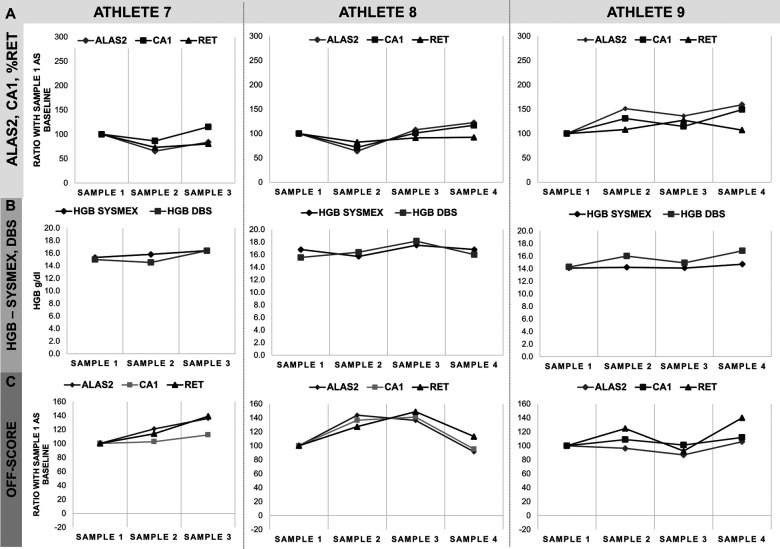
Monitoring of three athletes from skiing. **(A)** ALAS2, CA1 and%RET data as a percentage of the first sample, considered as baseline value, and corresponding 100% on the *y*-axis. **(B)** Comparison of HGB-values obtained in Sysmex-analysis and as calculated on the DBS. HGB data are represented in *y*-axis as g/dl. **(C)** Calculated OFF-score for all markers based on HGB, %RET, ALAS2 and CA1 data. ALAS2 is represented in dark grey diamonds (♦), CA1 in light grey squares (▪), %RET in black triangles (▴), HGB SYSMEX in black diamonds (♦) and HGB DBS in light grey squares (▪). Athlete 7: Skiing/Cross-country (male); Athlete 8: Skiing/Cross-country (male); Athlete 9: Skiing/Cross-country (male). Samples were collected in monthly intervals.

**Figure 5:**
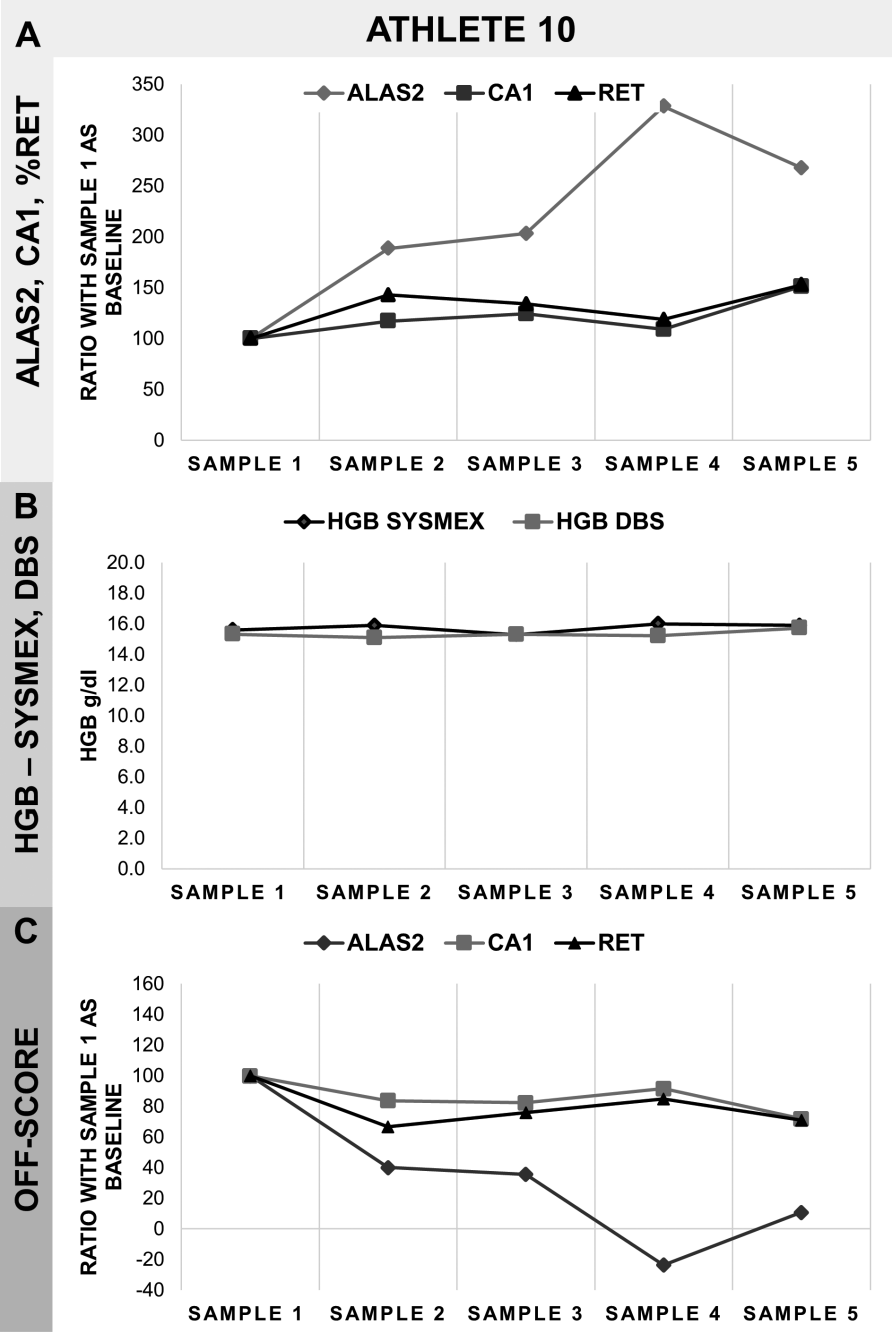
Monitoring of on athlete from ski-mountaineering. **(A)** ALAS2, CA1 and%RET data as a percentage of the first sample, considered as baseline value, and corresponding 100% on the *y*-axis. **(B)** Comparison of HGB-values obtained in Sysmex-analysis and as calculated on the DBS. HGB data are represented in *y*-axis as g/dl. **(C)** Calculated OFF-score for all markers based on HGB,%RET, ALAS2 and CA1 data. ALAS2 is represented in dark grey diamonds (♦), CA1 in light grey squares (▪), %RET in black triangles (▴), HGB SYSMEX in black diamonds (♦) and HGB DBS in light grey squares (▪). Samples were collected in monthly intervals.

Reticulocyte percentage (%RET) and hemoglobin concentration (HGB) data were obtained by Sysmex analysis on EDTA whole blood samples, while ALAS2 and CA1 levels and hemoglobin concentration were measured by RT-qPCR analysis and absorbance on DBS, respectively. Samples were collected from the athletes between 3 and 5 times (Table 1). Data were categorized by sport disciplines: cycling (road and track endurance) (Figure 2), athletics (long and middle distance) (Figure 3), cross-country skiing (Figure 4), and mountaineering (Figure 5). Most athlete's samples exhibited low variability of the selected parameters across different sample collection sessions, with amplitudes between 50% and 150% (Figures 2–4), which could be considered natural variation as demonstrated in (19). No significant difference related to variability were observed between sports and genders. From the individual athlete's perspective, most passports exhibited RNA-biomarker variations that mirrored the trends in%RET values. This comparison was observed in previous studies (17, 19–21, 23, 25). Measurement of HGB values with Sysmex XN followed the same trend as valued from DBS. Nonetheless, Sysmex value lower than DBS values were observed (see Athlete 1). This difference was already described (21, 27, 28).

However, one individual (ski mountaineering) demonstrated an increase in ALAS2 values at point 4 and maintained relatively high values from the baseline (Figure 5). The passport of this athlete also exhibited the highest amplitude, reaching a maximum of 350% higher values from the baseline. Furthermore, samples 2 to 5 were normalized against the sample 1. Thus, it is also possible that sample 1 was unusually low, which could have exaggerated the increases observed in the other samples.

An increase in ALAS2 may be linked to erythropoiesis stimulation, which can result from various factors such as blood loss, blood withdrawal, or doping with substances or medication such as erythropoiesis-stimulating agents (ESAs). Previously, it has been shown that altitude seemed less a confounding factor for RNA-biomarkers, especially when they are combined (17). Moreover, the athlete did not declare high altitude training/staying on doping control form that could explain this change. Regarding other known confounding factors, iron injection (not forbidden in sport) has no impact on RNA-biomarkers (29). The assay was performed in two independent analyses to exclude methodological variation.

Stimulation by recombinant EPOs, which can be detected by ALAS2 and CA1, seems unlikely as CA1 levels did not increase (17). Other compounds capable of stimulating erythropoiesis, such as Hypoxia Inducible Factor (HIF) stabilizers like vadadustat or roxadustat, may also be involved, although their effects on the RNA biomarkers studied here are not yet known (30). HiF stabilizers action mimic altitude and hypoxia impact on erythropoiesis (31). Since altitude seemed less a cofounding factor for RNA-biomarkers, further investigations such as clinical studies are needed to test this hypothesis. However, since HIF stabilizers are detected in urine samples, testing for these prohibited substances is recommended (32, 33). The hypothesis of blood withdrawal, which is not categorized as prohibited method by WADA, could also be considered. Research has shown that the peak of decrease in hemoglobin (HGB) occurs one week after the withdrawal of 450 ml of blood (34). However, the athlete's passport showed that HGB levels remained stable (Figure 5). Additionally, low-volume blood withdrawal has been found to have no significant impact on HGB concentration (35). Therefore, a recommendation could be given to tighter follow-up of this athlete.

This study has a limitation. For individual follow-up, 3–5 samples were collected and analyzed from each athlete. This number was determined by the project timeline and the planned sample collection schedule for the selected athletes. While this provided valuable data, a larger sample size could offer a more comprehensive dataset for analysis. Future studies will include a greater number of samples to enhance the depth and accuracy of the findings.

## Conclusion and perspectives

Testing RNA-biomarkers such as ALAS2 and CA1 method on routine samples highlight the feasibility of implementing the method in the process of analyzing EDTA samples. These data demonstrate that our proposed practical application, tested on routine ABP blood samples, can be successfully implemented without significant logistical or analytical constraints.

The RNA-biomarker method utilizes a PCR instrument, known for its user-friendly operation and minimal expertise requirements. PCR technology has been routinely employed in clinical settings for decades, ensuring its robustness and reliability. The cost of a standard RT-qPCR instrument ranges from 30,000 to 50,000 CHF, making the PCR reaction both affordable and efficient. Additionally, PCR instruments are gradually entering to anti-doping laboratories, particularly for gene doping detection (36). Consequently, RNA-biomarkers method could be available in every anti-doping laboratory.

Samples for RNA-biomarker detection are derived from routine EDTA samples, which are spotted on DBS cards. Therefore, good communication between the ADO and laboratory is essential for pre-determination of the samples (athletes) for this approach, if not applied to all samples. Nonetheless, the systemic spotting of EDTA blood samples onto DBS cards is feasible also for the laboratory routine, as it is not necessary to be performed immediately after the Sysmex analysis but could be adapted depending on the laboratory routine. The added value for the ABP assessment is that following the passport review, the APMU and experts can recommend RNA-biomarker detection as a complementary analysis to aid in the interpretation of suspicious ABP profiles. For a future perspective, a follow-up study could investigate the detection of RNA-biomarkers directly from capillary DBS (ex. Tasso M20 device) and compare the results with the corresponding RET% profile (37).

In conclusion, incorporating ALAS2 and CA1 with %RET, or even integrating them into the ABP, presents promising opportunities for enhancing anti-doping efforts in the future.

## Data Availability

The raw data supporting the conclusions of this article will be made available by the authors, without undue reservation.
